# *N*-(2-Hydroxy­phen­yl)-4-nitro­phthalimide

**DOI:** 10.1107/S1600536808025920

**Published:** 2008-08-16

**Authors:** Shahirah Mansor, Norzalida Zakaria, Azhar Ariffin, Seik Weng Ng

**Affiliations:** a Department of Chemistry, University of Malaya, 50603 Kuala Lumpur, Malaysia

## Abstract

Mol­ecules of the title compound, C_14_H_8_N_2_O_5_, are linked by a hydr­oxy–amide O—H⋯O hydrogen bond into a linear chain. The hydr­oxy group is disordered over two positions of the benzene ring in an approximate 0.57:0.43 ratio.

## Related literature

For literature on the hydrolysis of *N*-substituted phthalimides, see: Sim *et al.* (2006 ▶; 2007 ▶).
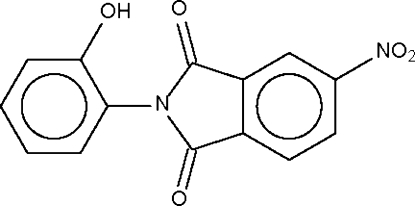


## Experimental

### 

#### Crystal data

C_14_H_8_N_2_O_5_
*M* *_r_* = 284.22
Orthorhombic, 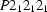

*a* = 7.1114 (2) Å
*b* = 11.7646 (3) Å
*c* = 14.5304 (4) Å
*V* = 1215.65 (6) Å^3^
*Z* = 4
Mo *K*α radiation
μ = 0.12 mm^−1^
*T* = 100 (2) K
0.32 × 0.06 × 0.06 mm

#### Data collection

Bruker SMART APEX diffractometer
Absorption correction: none
13791 measured reflections
1618 independent reflections
1356 reflections with *I* > 2σ(*I*)
*R*_int_ = 0.087

#### Refinement

*R*[*F* ^2^ > 2σ(*F* ^2^)] = 0.049
*wR*(*F* ^2^) = 0.142
*S* = 1.04
1618 reflections
199 parameters
2 restraints
H-atom parameters constrained
Δρ_max_ = 0.36 e Å^−3^
Δρ_min_ = −0.28 e Å^−3^

### 

Data collection: *APEX2* (Bruker, 2007 ▶); cell refinement: *SAINT* (Bruker, 2007 ▶); data reduction: *SAINT*; program(s) used to solve structure: *SHELXS97* (Sheldrick, 2008 ▶); program(s) used to refine structure: *SHELXL97* (Sheldrick, 2008 ▶); molecular graphics: *X-SEED* (Barbour, 2001 ▶); software used to prepare material for publication: *publCIF* (Westrip, 2008 ▶).

## Supplementary Material

Crystal structure: contains datablocks global, I. DOI: 10.1107/S1600536808025920/lh2681sup1.cif

Structure factors: contains datablocks I. DOI: 10.1107/S1600536808025920/lh2681Isup2.hkl

Additional supplementary materials:  crystallographic information; 3D view; checkCIF report

## Figures and Tables

**Table 1:** Hydrogen-bond geometry (Å, °)

*D*—H⋯*A*	*D*—H	H⋯*A*	*D*⋯*A*	*D*—H⋯*A*
O1—H1⋯O3^i^	0.84	1.99	2.747 (4)	149
O1′—H1′⋯O2^ii^	0.84	2.23	2.779 (4)	123

Symmetry codes: (i) 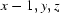; (ii) 
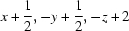
.

**Fig. 1.**
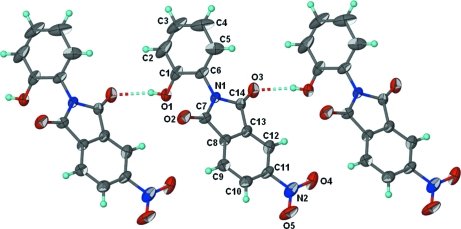
Thermal ellipsoid plot (Barbour, 2001) of O–H···O hydrogen-bonded structure of C14H8N2O3 at the 70% probability level. Dashed lines denote the intermolecular hydrogen bonds. Hydrogen atoms are drawn as spheres of arbitrary radius. Only the major component of disorder is shown.

## supplementary crystallographic information

### Comment

The title compound (Fig. 1) was synthesized for studies on intramolecular general base (IGB) and intramolecular general acid (IGA) catalysis in the hydrolysis of *N*-substitutedphthalimide (Sim *et al.*, 2006; 2007).

### Experimental

4-Nitrophthalic anhydride (5.0 g, 26 mmol) and *o*-hydroxyaniline (3.4 g, 31 mmol) were heated in glacial acetic acid (15 mol) for 4 h at 393–401 K. The reaction was shown to be complete by thin layer chromatography. The mixture was poured into water. The yellow solid was collected in 90% yield; purification was effected by recrystallization from chloroform.

### Refinement

Carbon-bound H-atoms were placed in calculated positions (C—H 0.95 Å) and were included in the refinement in the riding model approximation, with *U*(H) set to 1.2U_eq_(C). The hydroxy group is disordered over two positions on the phenylene ring; the disorder refined to a 0.571 (1):429 (1) ratio.

### Crystal data

**Table:**

C_14_H_8_N_2_O_5_	*F*_000_ = 584
*M**_r_* = 284.22	*D*_x_ = 1.553 Mg m^−^^3^
Orthorhombic, *P*2_1_2_1_2_1_	Mo *K*α radiation λ = 0.71073 Å
Hall symbol: P 2ac 2ab	Cell parameters from 2147 reflections
*a* = 7.1114 (2) Å	θ = 2.8–23.8º
*b* = 11.7646 (3) Å	µ = 0.12 mm^−^^1^
*c* = 14.5304 (4) Å	*T* = 100 (2) K
*V* = 1215.65 (6) Å^3^	Prism, yellow
*Z* = 4	0.32 × 0.06 × 0.06 mm

### Data collection

**Table:**

Bruker SMART APEX diffractometer	1356 reflections with *I* > 2σ(*I*)
Radiation source: fine-focus sealed tube	*R*_int_ = 0.087
Monochromator: graphite	θ_max_ = 27.5º
*T* = 100(2) K	θ_min_ = 2.2º
ω scans	*h* = −9→9
Absorption correction: None	*k* = −15→15
13791 measured reflections	*l* = −18→18
1618 independent reflections	

### Refinement

**Table:**

Refinement on *F*^2^	Secondary atom site location: difference Fourier map
Least-squares matrix: full	Hydrogen site location: inferred from neighbouring sites
*R*[*F*^2^ > 2σ(*F*^2^)] = 0.049	H-atom parameters constrained
*wR*(*F*^2^) = 0.142	*w* = 1/[σ^2^(*F*_o_^2^) + (0.0803*P*)^2^ + 0.3691*P*] where *P* = (*F*_o_^2^ + 2*F*_c_^2^)/3
*S* = 1.05	(Δ/σ)_max_ = 0.001
1618 reflections	Δρ_max_ = 0.36 e Å^−^^3^
199 parameters	Δρ_min_ = −0.28 e Å^−^^3^
2 restraints	Extinction correction: none
Primary atom site location: structure-invariant direct methods	

### Fractional atomic coordinates and isotropic or equivalent isotropic displacement parameters (Å^2^)

**Table:**

	*x*	*y*	*z*	*U*_iso_*/*U*_eq_	Occ. (<1)
O1	0.3544 (4)	0.2939 (3)	0.8037 (3)	0.0308 (10)	0.571 (3)
H1	0.2490	0.3065	0.7797	0.046*	0.571 (3)
O1'	0.8498 (5)	0.4876 (3)	0.9237 (4)	0.0287 (13)	0.429 (3)
H1'	0.9140	0.4284	0.9162	0.043*	0.429 (3)
O2	0.4761 (4)	0.1944 (2)	0.97117 (17)	0.0362 (6)	
O3	0.9695 (3)	0.3018 (2)	0.78879 (18)	0.0347 (6)	
O4	1.3585 (4)	−0.0916 (2)	0.8641 (2)	0.0455 (8)	
O5	1.1873 (5)	−0.2264 (2)	0.9238 (2)	0.0533 (9)	
N1	0.7000 (4)	0.2751 (2)	0.87514 (18)	0.0217 (6)	
N2	1.2106 (5)	−0.1285 (3)	0.8959 (2)	0.0383 (8)	
C1	0.4328 (5)	0.3850 (3)	0.8206 (2)	0.0307 (8)	
H1A	0.3676	0.3163	0.8080	0.037*	0.429 (3)
C2	0.3486 (7)	0.4888 (4)	0.8022 (3)	0.0493 (12)	
H2	0.2230	0.4915	0.7800	0.059*	
C3	0.4465 (8)	0.5871 (4)	0.8161 (3)	0.0537 (13)	
H3	0.3904	0.6578	0.8006	0.064*	
C4	0.6260 (9)	0.5852 (3)	0.8525 (3)	0.0543 (14)	
H4	0.6931	0.6542	0.8616	0.065*	
C5	0.7071 (6)	0.4825 (3)	0.8755 (3)	0.0390 (9)	
H5A	0.8276	0.4806	0.9037	0.047*	0.571 (3)
C6	0.6120 (5)	0.3820 (3)	0.8574 (2)	0.0271 (7)	
C7	0.6224 (5)	0.1884 (3)	0.9290 (2)	0.0237 (7)	
C8	0.7584 (5)	0.0922 (3)	0.9252 (2)	0.0236 (7)	
C9	0.7433 (5)	−0.0146 (3)	0.9637 (2)	0.0287 (7)	
H9	0.6348	−0.0371	0.9972	0.034*	
C10	0.8946 (5)	−0.0877 (3)	0.9510 (2)	0.0305 (8)	
H10	0.8915	−0.1625	0.9756	0.037*	
C11	1.0478 (5)	−0.0508 (3)	0.9029 (2)	0.0270 (7)	
C12	1.0651 (5)	0.0568 (3)	0.8618 (2)	0.0276 (7)	
H12	1.1733	0.0793	0.8281	0.033*	
C13	0.9122 (4)	0.1269 (3)	0.8746 (2)	0.0244 (7)	
C14	0.8758 (4)	0.2439 (3)	0.8398 (2)	0.0226 (7)	

### Atomic displacement parameters (Å^2^)

**Table:**

	*U*^11^	*U*^22^	*U*^33^	*U*^12^	*U*^13^	*U*^23^
O1	0.021 (2)	0.032 (2)	0.039 (2)	0.0014 (18)	−0.0039 (18)	0.000 (2)
O1'	0.025 (3)	0.023 (2)	0.039 (3)	−0.003 (2)	−0.014 (2)	0.000 (2)
O2	0.0371 (14)	0.0391 (13)	0.0322 (14)	0.0103 (12)	0.0161 (11)	0.0066 (11)
O3	0.0246 (12)	0.0454 (15)	0.0341 (13)	0.0017 (12)	0.0003 (11)	0.0114 (12)
O4	0.0307 (14)	0.0623 (19)	0.0435 (15)	0.0172 (13)	0.0023 (13)	−0.0015 (14)
O5	0.070 (2)	0.0364 (14)	0.0534 (18)	0.0275 (15)	0.0170 (17)	0.0133 (14)
N1	0.0209 (12)	0.0235 (13)	0.0206 (13)	0.0047 (10)	−0.0020 (11)	0.0006 (11)
N2	0.0418 (17)	0.0448 (18)	0.0285 (16)	0.0170 (15)	0.0059 (14)	−0.0016 (15)
C1	0.0310 (17)	0.0420 (19)	0.0191 (15)	0.0149 (16)	0.0059 (13)	0.0048 (15)
C2	0.053 (3)	0.058 (3)	0.036 (2)	0.035 (2)	0.017 (2)	0.025 (2)
C3	0.081 (3)	0.048 (3)	0.032 (2)	0.042 (3)	0.021 (2)	0.0186 (19)
C4	0.109 (4)	0.0286 (19)	0.0250 (19)	0.013 (2)	0.011 (3)	0.0013 (16)
C5	0.066 (3)	0.0290 (17)	0.0217 (18)	0.0023 (18)	−0.0074 (19)	0.0015 (15)
C6	0.0371 (17)	0.0255 (15)	0.0186 (15)	0.0124 (14)	0.0028 (14)	0.0026 (13)
C7	0.0313 (16)	0.0246 (14)	0.0152 (14)	0.0044 (13)	−0.0018 (13)	−0.0005 (12)
C8	0.0268 (15)	0.0257 (15)	0.0184 (15)	0.0057 (12)	0.0005 (13)	−0.0024 (13)
C9	0.0309 (16)	0.0302 (16)	0.0250 (17)	0.0010 (14)	0.0018 (14)	−0.0009 (14)
C10	0.0351 (17)	0.0284 (17)	0.0280 (17)	0.0038 (14)	−0.0024 (15)	−0.0038 (14)
C11	0.0324 (16)	0.0288 (16)	0.0198 (15)	0.0129 (14)	−0.0041 (13)	−0.0051 (14)
C12	0.0255 (15)	0.0382 (18)	0.0191 (15)	0.0056 (14)	−0.0033 (13)	−0.0003 (14)
C13	0.0229 (14)	0.0288 (16)	0.0214 (14)	0.0054 (12)	−0.0057 (13)	−0.0038 (14)
C14	0.0206 (14)	0.0279 (15)	0.0194 (15)	0.0014 (12)	−0.0040 (12)	0.0012 (13)

### Geometric parameters (Å, °)

**Table:**

O1—C1	1.233 (5)	C3—C4	1.382 (8)
O1—H1	0.8400	C3—H3	0.9500
O1'—C5	1.234 (5)	C4—C5	1.380 (6)
O1'—H1'	0.8400	C4—H4	0.9500
O2—C7	1.210 (4)	C5—C6	1.388 (5)
O3—C14	1.207 (4)	C5—H5A	0.9500
O4—N2	1.228 (4)	C7—C8	1.490 (4)
O5—N2	1.232 (4)	C8—C9	1.380 (5)
N1—C14	1.401 (4)	C8—C13	1.379 (4)
N1—C7	1.399 (4)	C9—C10	1.389 (5)
N1—C6	1.428 (4)	C9—H9	0.9500
N2—C11	1.478 (4)	C10—C11	1.366 (5)
C1—C6	1.382 (5)	C10—H10	0.9500
C1—C2	1.386 (5)	C11—C12	1.405 (5)
C1—H1A	0.9500	C12—C13	1.377 (4)
C2—C3	1.365 (8)	C12—H12	0.9500
C2—H2	0.9500	C13—C14	1.490 (5)
			
C1—O1—H1	109.5	C1—C6—C5	120.1 (3)
C5—O1'—H1'	109.5	C1—C6—N1	119.8 (3)
C14—N1—C7	111.5 (3)	C5—C6—N1	120.1 (3)
C14—N1—C6	123.7 (3)	O2—C7—N1	125.5 (3)
C7—N1—C6	124.8 (3)	O2—C7—C8	128.4 (3)
O4—N2—O5	124.7 (3)	N1—C7—C8	106.1 (3)
O4—N2—C11	118.6 (3)	C9—C8—C13	123.1 (3)
O5—N2—C11	116.7 (3)	C9—C8—C7	128.8 (3)
O1—C1—C6	118.1 (3)	C13—C8—C7	108.1 (3)
O1—C1—C2	122.1 (4)	C8—C9—C10	116.8 (3)
C6—C1—C2	119.7 (4)	C8—C9—H9	121.6
C6—C1—H1A	120.1	C10—C9—H9	121.6
C2—C1—H1A	120.1	C11—C10—C9	119.2 (3)
C3—C2—C1	119.8 (4)	C11—C10—H10	120.4
C3—C2—H2	120.1	C9—C10—H10	120.4
C1—C2—H2	120.1	C10—C11—C12	125.0 (3)
C2—C3—C4	121.0 (4)	C10—C11—N2	117.6 (3)
C2—C3—H3	119.5	C12—C11—N2	117.3 (3)
C4—C3—H3	119.5	C13—C12—C11	114.4 (3)
C5—C4—C3	119.5 (5)	C13—C12—H12	122.8
C5—C4—H4	120.2	C11—C12—H12	122.8
C3—C4—H4	120.2	C12—C13—C8	121.4 (3)
O1'—C5—C4	116.1 (4)	C12—C13—C14	130.2 (3)
O1'—C5—C6	123.4 (3)	C8—C13—C14	108.4 (3)
C4—C5—C6	119.7 (4)	O3—C14—N1	124.8 (3)
C4—C5—H5A	120.1	O3—C14—C13	129.3 (3)
C6—C5—H5A	120.1	N1—C14—C13	105.8 (3)
			
O1—C1—C2—C3	−175.3 (4)	C13—C8—C9—C10	1.3 (5)
C6—C1—C2—C3	3.3 (5)	C7—C8—C9—C10	−178.6 (3)
C1—C2—C3—C4	−3.1 (6)	C8—C9—C10—C11	0.5 (5)
C2—C3—C4—C5	−0.3 (6)	C9—C10—C11—C12	−1.6 (5)
C3—C4—C5—O1'	−166.2 (4)	C9—C10—C11—N2	176.2 (3)
C3—C4—C5—C6	3.5 (6)	O4—N2—C11—C10	−170.1 (3)
O1—C1—C6—C5	178.5 (4)	O5—N2—C11—C10	9.1 (5)
C2—C1—C6—C5	−0.1 (5)	O4—N2—C11—C12	7.9 (5)
O1—C1—C6—N1	−0.4 (5)	O5—N2—C11—C12	−172.9 (3)
C2—C1—C6—N1	−179.1 (3)	C10—C11—C12—C13	0.9 (5)
O1'—C5—C6—C1	165.7 (4)	N2—C11—C12—C13	−177.0 (3)
C4—C5—C6—C1	−3.2 (5)	C11—C12—C13—C8	1.0 (5)
O1'—C5—C6—N1	−15.3 (6)	C11—C12—C13—C14	−177.8 (3)
C4—C5—C6—N1	175.7 (3)	C9—C8—C13—C12	−2.1 (5)
C14—N1—C6—C1	124.8 (3)	C7—C8—C13—C12	177.8 (3)
C7—N1—C6—C1	−54.7 (4)	C9—C8—C13—C14	176.9 (3)
C14—N1—C6—C5	−54.1 (4)	C7—C8—C13—C14	−3.2 (3)
C7—N1—C6—C5	126.3 (4)	C7—N1—C14—O3	177.4 (3)
C14—N1—C7—O2	176.5 (3)	C6—N1—C14—O3	−2.1 (5)
C6—N1—C7—O2	−3.9 (5)	C7—N1—C14—C13	0.0 (3)
C14—N1—C7—C8	−1.8 (3)	C6—N1—C14—C13	−179.6 (3)
C6—N1—C7—C8	177.8 (3)	C12—C13—C14—O3	3.7 (6)
O2—C7—C8—C9	4.8 (6)	C8—C13—C14—O3	−175.3 (3)
N1—C7—C8—C9	−177.0 (3)	C12—C13—C14—N1	−179.0 (3)
O2—C7—C8—C13	−175.1 (3)	C8—C13—C14—N1	2.1 (3)
N1—C7—C8—C13	3.1 (3)		

### Hydrogen-bond geometry (Å, °)

**Table:**

*D*—H···*A*	*D*—H	H···*A*	*D*···*A*	*D*—H···*A*
O1—H1···O3^i^	0.84	1.99	2.747 (4)	149
O1'—H1'···O2^ii^	0.84	2.23	2.779 (4)	123

Symmetry codes: (i) *x*−1, *y*, *z*; (ii) *x*+1/2, −*y*+1/2, −*z*+2.
